# A Retrospective Study on Pathologic Features and Racial Disparities in Prostate Cancer

**DOI:** 10.1155/2011/239460

**Published:** 2011-10-31

**Authors:** Steven A. Bigler, Charles R. Pound, Xinchun Zhou

**Affiliations:** ^1^Department of Pathology, University of Mississippi Medical Center, Jackson, MS 39216, USA; ^2^Department of Surgery, University of Mississippi Medical Center, Jackson, MS 39216, USA

## Abstract

We reviewed more than 3,000 pathology reports on prostate cancer-related surgical specimens and analyzed racial disparities in histological and clinical features at the time of initial biopsy, diagnosis of prostate cancer, and prostatectomy, as well as in characteristics of tumor evolution between African American and Caucasian patients. As compared to Caucasians, African American patients had younger age, higher cancer detection rate, higher Gleason score of prostate cancer, and more bilateral involvement of the prostate. African Americans also had larger prostates, greater volume of tumor, and more positive margins. The diagnosis of HGPIN or ASAP in prostate biopsies and African American race conferred an increased risk of diagnosis of prostate cancer. The interval between prior noncancerous biopsy and the subsequent biopsy with diagnosis of prostate cancer was shorter in men with HGPIN, with ASAP, or of African American race.

## 1. Introduction

African American race along with age and family history are well-established risk factors for prostate cancer [1]. A better understanding of the racial disparities in prostate cancer between African Americans and Caucasians is crucial to elucidating the pathogenesis of this disease, developing rational diagnostic and screening strategies, and facilitating discovery of new interventions for prevention and treatment of prostate cancer. That African Americans have higher incidence and mortality rates than Caucasians is clear from epidemiological studies using nationwide databases such as the SEER database maintained by the National Cancer Institute [2]. Many studies have demonstrated that there was higher detection rates of prostate cancer in biopsies from African Americans compared to Caucasians [3–6], while other studies suggest that race might not be independently associated with a positive biopsy in men suspected of having prostate cancer [7–9]. It has been commonly accepted that high-grade prostatic intraepithelial neoplasia (HGPIN) is an important histological precursor of prostate cancer since it was precisely characterized by McNeal and Bostwick [10], and atypical small acinar proliferation (ASAP) is a histological finding associated with a high rate of current carcinoma not sampled at the time of biopsy [11]. Both lesions are generally believed to be important predictors for prostate cancer on rebiopsy [12–14]. Racial differences in prevalence of these important histological patterns have been demonstrated in prior studies [4, 15], but the significance of such distinctions and application of this knowledge are not agreed upon. For example, the role of race in developing strategies for rebiopsy in men with HGPIN is controversial [6, 16]. Thus, the influence of these characteristic lesions on the evolution of prostate cancer needs to be further studied. 

There are many potential reasons for these discrepancies between studies of racial differences in histological features associated with prostate cancer, including lack of reproducibility in the diagnosis of HGPIN or ASAP [13]. One of the most important limitations in our understanding in this area is that African Americans remain underrepresented in most cancer studies [17–19]. There is a need for additional studies from regions and institutions with populations which include a substantial proportion of African Americans. The population of Mississippi is comprised of a relatively stable racial/ethnic mix, and most individuals in the state identify themselves as either African American or Caucasian (~98%) with a nearly balanced proportion [20]. Less than 1% of people from Mississippi were reported as more than one race in census data [21]. Importantly, incidence and mortality rates from prostate cancer in Mississippi rank among the nation's highest [22]. The current study will retrospectively analyze the pathology reports and clinical data for African American and Caucasian patients with prostatic diseases from the Mississippi region. We will emphasize the racial disparities in pathological and clinical features at the time of initial biopsy, diagnosis of prostate cancer, and prostatectomy, as well as in the characteristics of tumor evolution. 

## 2. Materials and Methods

### 2.1. Patient Population, Data Collection, and Categorization

All patients were registered at the University of Mississippi Medical Center (UMMC), a state owned tertiary care hospital between 1989 and 2009. A large majority of the patients were from Mississippi. Records of patients with surgical specimens from the prostate or other sites with metastatic prostate cancer were reviewed for this study. Of 2,403 patients, 1261 (52.48%) were African American, and 1019 (42.41%) were Caucasian, 9 (0.4%) were other races including Asian, Hispanic, and American Indian. The remaining 114 (4.7%) were patients without racial/ethnic information recorded. 

Pathology reports for each surgical specimen were reviewed. Patient medical history and laboratory data were obtained from electronic databases at the UMMC. Most of the pathologic diagnoses were made by one of the authors (SB). Cases were categorized as benign, HGPIN, ASAP, primary prostate cancer, and metastatic prostate cancer. Specimens with more than one diagnosis were included in only one group by assigning them to the highest category present in descending order from metastatic prostate cancer, primary prostate cancer, ASAP and HGPIN to benign. Twenty-three surgical specimens were excluded from the study: 6 with benign diagnoses, including 3 adenomas, 1 leiomyoma and 2 papillomas, and 17 with malignant diagnoses, including 1 adenoid basal cell tumor, 1 adenocarcinoma unspecified, 2 sarcomas, 2 squamous cell carcinomas, 1 stromal and epithelial tumor, and 10 transitional cell carcinomas. Racial differences were analyzed only between African American and Caucasian patients due to limited numbers from other racial groups. 

### 2.2. Prostatic Biopsy

Indications for an initial prostatic biopsy included elevation of serum PSA (>4 ng/mL), abnormal digital rectum examination (DRE), and clinical manifestations of urinary outlet obstruction. Repeat biopsies were mostly performed for patients who had previously negative biopsy for prostate cancer in whom there was high clinical suspicion for prostate cancer. Included in this study, approximately 61% biopsies were standard sextant biopsies; the other biopsy cases had a variable number of additional biopsy cores. During the study period, a total of 2,248 prostatic biopsies were performed by multiple urologists. All criteria and procedures and protocols were the same for patients from African American and Caucasian populations.

### 2.3. Prostatectomy

In this study, a total of 442 prostates were surgically removed. Among these, 361 (81.7%) cases were radical retropubic prostatectomies, and 81 (18.3%) patients underwent cystoprostatectomy. When the entire prostate (or with urinary bladder in cystoprostatectomy) was removed, it was immediately fixed in formalin. The prostate, after the removal of seminal vesicles, was weighed in grams at the time of gross examination. A few prostates were not weighed (mostly attached with diseased urinary bladder) and their weights were estimated by three dimensional measurements with the formula: weight (grams) = length (cm) × height (cm) × width (cm) × 3.14/6 [23]. The entire prostate was sectioned at 3 mm thickness and processed and stained with hematoxylin eosin routinely for pathology study. The percentage of tumor, the proportion of tissue area occupied by tumor in the entire gland, was estimated by visual inspection (mostly by SB). The tumor volume was calculated using the formula: tumor volume (cm^3^) = % of tumor × weight of prostate (grams). The surgical and pathological procedures were exactly the same for patients from African American and Caucasian populations. 

### 2.4. Parameters in the Evolution of Prostate Cancer

In order to compare the differences in the evolution of prostate cancer between African American and Caucasians, the following parameters were set for comparison: (1) detection rate and features of prostate cancer in repeat biopsies, (2) laterality of prostate pathology in initial biopsy, and (3) time interval between two consecutive prostatic biopsies. The time interval for evolution of prostate cancer was calculated from the time of the latest negative biopsy to the time of the earliest positive sampling in patients who were eventually diagnosed as prostate cancer by repeat sampling. 

### 2.5. Statistical Analysis

Chi-square test was used in comparison of rates or percentages; Wilcoxon-Mann-Whitney test was used to compare medians; Student's *t*-test was used in comparison of means; exact binomial probabilities calculation was used in testing binomial distributions. 

## 3. Results

A total of 3,315 pathology reports from 2,403 patients have been reviewed. Of 3,315 surgical specimens from the prostate or other sites related to prostate tumors, 1480 (44.7%) were diagnosed as benign changes including diagnoses of normal, hyperplasia, and prostatitis; 252 (7.6%) as HGPIN; 90 (2.7%) as ASAP; 1,435 as primary prostate cancer (43.7%); 35 (1.06%) as metastatic prostate cancer.

### 3.1. Racial Disparities in Detection Rate, Age, PSA Level, and Tumor Grade at the Initial Biopsy

African Americans had 1,230 biopsies including 1,012 initial biopsies and 218 repeat biopsies from 147 patients; Caucasian had 911 biopsies including 765 initial biopsies, and 146 repeat biopsies from 121 patients. At the time of the initial biopsy, the age was significantly younger and the PSA level was significantly higher in African American than in Caucasian patients (mean age: 62 versus 63.8, *P* < 0.0001, median PSA: 8 ng/mL versus 5.8 ng/mL, *P* < 0.0001). As shown in Table 1, the detection rate of prostate cancer on initial biopsy was substantially higher in African American patients than in Caucasian patients (50.3% versus 38.3%, *P* < 0.0001), representing a relative risk of 1.31. Conversely, benign diagnoses excluding HGPIN, and ASAP on the initial biopsy were less common in African Americans than Caucasians (37.2% versus 48.4%, *P* = 0.001). There was no significant difference in the detection rate of either HGPIN or ASAP between African Americans and Caucasians. The mean age at the time of initial prostate biopsy was 1 to 2.5 years younger in African American patients regardless of the diagnosis. In patients with diagnoses of benign, HGPIN and ASAP, there was no significant difference in serum PSA level between the two races. The difference in PSA serum levels between patients with a benign diagnosis and patients with a diagnosis of cancer was much more dramatically increased in African American men (5.7 ng/mL versus 11.6 ng/mL, a 103% difference) than in Caucasian patients (5.5 ng/mL versus 7 ng/mL, a 27% difference).

### 3.2. Racial Disparities in Variables at the Time of Diagnosis of Prostate Cancer

The racial differences in clinical and pathologic variables at the time of diagnosis for prostate cancer are outlined in Table 2. African Americans not only had significantly higher detection rates of prostate cancer (49.2% versus 40.8%, *P* < 0.0001), but they were also younger (63.1 versus 64.7, *P* < 0.0081), with higher Gleason scores (6.9 versus 6.3, *P* < 0.0001), and with higher serum PSA levels (11.1 versus 7, *P* < 0.0001). Most prostate cancers were diagnosed at the time of the initial prostate biopsy in both races. This was especially true in African Americans, of 620 African American patients with prostate cancer, 509 (82.1%) were diagnosed at the initial prostate biopsy, which was significantly higher than the proportion of Caucasian cancer patients diagnosed at the time of the initial biopsy (293 out of 416, 70.5%, *P* < 0.0001). 

### 3.3. Racial Disparities in Pathologic Features in Radical Prostatectomy

Detailed pathologic features of prostatectomy and initial diagnostic reports were available for 185 African American and 152 Caucasian patients. The racial differences in pathologic features in radical prostatectomy and cystoprostatectomy specimens are summarized in Table 3. Caucasians had a higher proportion of cystoprostatectomy specimens than African American patients (27.2% versus 9.8%, *P* < 0.0001). In cystoprostatectomy specimens, the rate of incidental prostate cancer (primary purpose of surgically removal of the prostate was not for prostate cancer) in cystoprostatectomy was 39.1% in African Americans and 34.5% in Caucasians (no significant difference). It is worth noting that African American patients diagnosed with prostate cancer were almost 3 years younger than Caucasians at prostatectomy (58.7 versus 61.5, *P* = 0.0005). African American patients had a significantly higher rate of positive resection margins (33.5% versus 19.9%, *P* = 0.0005) than Caucasians. Although the median weight of the prostate was almost the same in the two races, African American patients had a significantly higher median percentage of tumors in the gland (15% versus 6.6%, *P* = 0.014) or more than 2 times of tumor volumes (7.4 cm^3^ versus 3 cm^3^, *P* = 0.012) as compared to Caucasians. The differences in other pathologic features, including Gleason score, were not significant in the prostatectomy specimens between the two races. 

### 3.4. Racial Disparities in Progression and Evolution of Prostate Cancer

**Figure 1:**
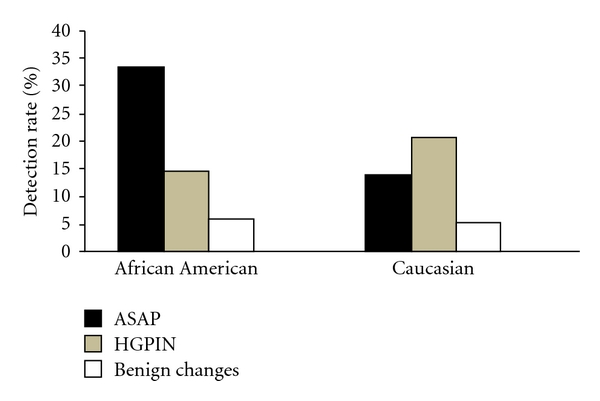
Comparison of prostate cancer detection rate in repeat sampling between African American and Caucasian.

In our study cohort, approximately 7–9% of the patients diagnosed with prostate cancer were identified on repeat prostate biopsy procedures (6.8% in African American and 9.1% in Caucasian). The detection rate of prostate cancer at the repeat biopsy varied with the diagnosis in previous biopsies and races. As shown in Figure 1, both HGPIN and ASAP were associated with increased detection rates for prostate cancer in subsequent biopsies. In African Americans, prostate cancer was detected by repeat sampling in 33% patients with ASAP diagnosed in previous biopsies; this rate was significantly higher than that in patients with PIN (14.7%, *P* = 0.01, OR = 2.9, 95% CI: 1.4–6.2) or only benign features (5.8%, *P* < 0.0001, OR = 8.1, 95% CI: 4.2–15.8) diagnosed in previous biopsies. The difference in detection rate of prostate cancer between patients with previous diagnosis of PIN and benign was also significant (14.7% versus 5.8%, *P* = 0.001, OR = 3.0, 95% CI: 1.6–4.8). In Caucasians, prostate cancer was detected by repeat sampling in 20.6% of patients with PIN diagnosed in previous biopsies; this rate was significantly higher than that in patients with ASAP (13.8%, *P* = 0.01, OR = 2.9, 95% CI: 1.4–6.2) or only benign features (5.1%, *P* < 0.0001, OR = 4.8, 95% CI: 2.7–8.7) diagnosed in previous biopsies. There was no statistical difference between the two races in the detection of prostate cancer by repeat sampling from patients with previous diagnoses of ASAP, PIN, and benign although prostate cancer detected by repeat sampling in patients with ASAP diagnosed in previous biopsy in African Americans was 2.4 times higher than that in Caucasians (33.3% versus 13.8%, *P* = 0.067, OR = 3.1, 95% CI: 1–10).

In African American patients in which there was clinical suspicious for prostate cancer, the time interval, which was calculated from the time of noncancer at the previous biopsy to the progression of prostate cancer at the subsequent biopsy, was 7 months for those with previous diagnosis of HGPIN or ASAP and 9 months for those with previous diagnosis of benign. In Caucasian patients, that time interval was 8 months for those with previous diagnosis of HGPIN or ASAP and 22.5 months for those with previous diagnosis of benign. These data suggest that prostate cancer might progress more rapidly in African Americans with persistently elevated PSA, especially those with previous diagnosis of benign, as compared to Caucasians. 

Information regarding laterality of prostate pathology diagnosed at initial biopsy was available for 969 patients, including 165 patients with HGPIN, 51 patients with ASAP, and 753 patients with prostate cancer. African Americans patients had higher percentages of bilateral distribution of all HGPIN, ASAP, and prostate cancer than Caucasians. The differences were significant in HGPIN (*P* = 0.004) and in prostate cancer (*P* < 0.0001). Although African Americans had a higher percentage of bilaterally distributed ASAP than Caucasians (23.3% versus 4.8%) with an odds ratio of 6.08 (Table 4), the difference was not statistically significant. These results suggest that prostate cancer was more advanced in African Americans at the time of initial biopsy than in Caucasians. 

In 142 unilaterally distributed HGPIN and ASAP, it was significantly more commonly detected on the left side (88 out of 142, 62% in left half of prostate, with a Z-ratio of +2.77, *P* = 0.0054). This left predominance remained similar in both lesions of HGPIN and ASAP and in both races. The significance of this phenomenon is not determined but may be due to the handedness of the physicians performing the biopsies. 

## 4. Discussion

This retrospective study demonstrates that, in this cohort of African American and Caucasian men undergoing biopsy or resection for prostate cancer, there are significant racial differences in clinical and pathologic parameters. African Americans were younger at the time of initial biopsy and at the time of cancer diagnosis. The detection rate for prostate cancer was nearly 30% higher in African American men undergoing an initial biopsy procedure than for Caucasian men, and the cancers detected on initial biopsy procedure were 70% more likely to be bilateral and with higher Gleason scores in African Americans. Prostate cancers in African Americans had greater volume and occupied a higher percentage of the prostate gland than cancers in Caucasians, and African Americans were more likely to have positive surgical resection margins at prostatectomy than Caucasians. If patients with positive surgical resection margins were accounted into non-organ-confined disease, African Americans had a significantly higher rate of non-organ-confined diseases at the time of prostatecotomy for cancer as compared to Caucasians (non-organ-confined cases was 43.4% in African American 32.9% in Caucasian, *P* = 0.044, data not shown in results). Of course, some positive surgical resection margins might be technically produced by transection of organ-confined tumors.

Cancer detection at the time of the initial prostate biopsy procedure was higher in both races in our study compared to reported rates in the medical literatures [24, 25]. The University of Mississippi Medical Center is the only allopathic medical school in Mississippi and is an important tertiary referral center for medical care. Mississippi, in addition to having a high proportion of African American citizens, is also a relatively poor state with lower per capita income and fewer physicians per capita than other states, which may contribute to lower rates of participation in health screening endeavors, including PSA screening. These factors may contribute to the higher rates of cancer detection on initial biopsy and the higher levels of PSA at initial diagnosis, compared to published data.

The focus of this review was to evaluate histological features of prostate tissue samples including biopsies and resections to identify racial differences and to correlate the differences with clinical factors associated with increased risk and poor prognosis for prostate cancer in African Americans. We were able to identify higher rates of prostate cancer precursors in African Americans and to show that both HGPIN and ASAP are important risk factors for subsequent diagnosis of adenocarcinoma in both races. In fact, the risk of subsequent cancer diagnosis for both HGPIN and ASAP was higher in African American patients than in Caucasian patients. This supports the concept that prostate cancer development in African Americans is pathogenetically similar to tumorigenesis in Caucasian patients and is associated with the same precursor lesions. Although African American patients were younger at the time of initial prostate biopsy and at the time of prostatectomy than Caucasian patients, their tumor volumes were greater, probably accounting for the more frequent positive margins, higher PSA levels, and higher frequency of bilateral cancer detection on biopsies. All of these findings indicate that significant prostate cancer develops at younger ages in African American men than Caucasians, which could have important implications for the development of evidence-based prostate cancer screening recommendations. In our opinion, it is important to inform the African American men in our communities about the risks of prostate cancer and encourage them to participate in appropriate screening programs. Whether African Americans should begin the screening process at a younger age needs to be further addressed. Health care providers need to be aware of these clinical differences and increased risk factors for African Americans.

Prostate cancer volumes in this review were estimates based on visual evaluation of representative, but thorough sampling and more accurate assessments of cancer volume are available from other studies. In our study, the median tumor volume in African Americans was approximately 2.5 times larger than that in Caucasians (7.4 cm^3^ versus 3 cm^3^); however, the median weight of the entire prostate gland was almost equal between the races (46 grams for African American, 45 grams for Caucasian). The ratio of prostate cancer volume between two races in this study was similar to that reported by Sanchez-Ortiz et al. [26], in which African Americans had 2.8 times larger prostate cancer volume than Caucasians (2.5 times in our study). However, the mean tumor volumes were much smaller (1.82 cm^3^ for African American, 0.72 cm^3^ for Caucasian) in their study of patients with nonpalpable T1 prostate cancer. Actually, the prostate cancer volume for Caucasians in our study (3 cm^3^) was close to that (3.4 cm^3^) reported by Moul et al. [27]. 

The reasons that African Americans develop prostate cancer at younger ages with higher Gleason scores and greater volumes than Caucasians remain unclear. Certainly, delays in diagnosis could be an important factor in African Americans having greater tumor volumes, but the fact that African Americans in our study were younger at the time of initial biopsy would suggest that biological factors could be responsible for these disparities. The differences between the races in androgen concentration, androgen sensitivity, diet, and cultures could be some of the potential factors of the racial disparities in prostate cancer. Perhaps African Americans have similar pathogenetic mechanisms in development of prostate cancer to Caucasians, but they have a faster growth rate and/or an earlier transformation to clinically significant prostate cancer as evidenced by Powell et al. [28]. 

## Figures and Tables

**Table 1:** Differences in percentages of diagnoses at the first biopsy between African American and Caucasian.

Diagnosis	African American (*n*)	Caucasian (*n*)	*P* value
Benign (%)	37.2 (372)	48.4 (370)	<0.0001^a^
Age (Year, mean ± SD)	60.9 ± 8.9 (376)	63.5 ± 9.2 (370)	<0.0001
Serum PSA (ng/mL, median)	5.7 (170)	5.5 (146)	>0.05
PIN (%)	9.1 (92)	9.9 (76)	>0.05
Age (Year, mean ± SD)	61 ± 7.7 (92)	62.4 ± 7 (76)	>0.05
Serum PSA (ng/mL, median)	6.7 (73)	5.5 (39)	>0.05
ASAP (%)	3.3 (33)	2.9 (22)	>0.05
Age (Year, mean ± SD)	60.5 ± 9 (33)	62.4 ± 7 (22)	>0.05
Serum PSA (ng/mL, median)	5.1 (21)	5.8 (13)	>0.05
Prostate cancer (%)	50.3 (509)	38.3 (293)	<0.0001^b^
Age (Year, mean ± SD)	63.2 ± 9 (509)	64.7 ± 9.1 (291)	0.027
Serum PSA (ng/mL, median)	11.6 (346)	7 (162)	<0.0001
Gleason's score (*n*/mean ± SD)	6.9 ± 1.5 (502)	6.3 ± 1.5 (289)	<0.0001

^a^: OR = 0.63, 95% CI: 0.52–0.76. ^b^: OR = 1.63, 95% CI: 1.35–1.97.

**Table 2:** Differences in features at time of diagnosis of Pca between African Americans and Caucasians.

Features	African American (*n*)	Caucasian (*n*)	*P* value
Prostate cancer detection rate (%)	49.2 (620)	40.8 (416)	<0.0001^a^
Age (year, mean±SD)	63.1 ± 9 (620)	64.7 ± 8.8 (414)	0.0081
Gleason score (mean±SD)	6.9 ± 1.6 (611)	6.3 ± 1.6 (410)	<0.0001
Serum PSA (ng/mL, median)	11.1 (404)	7 (213)	<0.0001
Diagnosed by biopsy (%)	88.9 (551)	79.6 (331)	<0.000^b^
First biopsy (%)	82.1 (509)	70.5 (293)	<0.0001^c^
Repeated biopsies (%)	6.8 (42)	9.1 (38)	>0.05
Diagnosed by TURP* (%)	6.5 (40)	9.6 (40)	0.074^d^
Diagnosed by prostatectomy (%)	4.7 (29)	10.8 (45)	<0.0001^e^

*TURP: Transurethral resection of prostate; ^a^: OR = 1.4, 95% CI: 1.19–1.66; ^b^: OR = 2.1, 95% CI: 1.45–2.89; ^c^: OR = 1.93, 95% CI: 1.44–2.58; ^d^: OR = 0.65, 95% CI: 0.41–1.02: ^e^: OR = 0.41, 95% CI: 0.25–0.65.

**Table 3:** Differences in clinical and pathological features at prostatectomy between African Americans and Caucasians.

Features	African American (*n*)	Caucasian (*n*)	*P* value
Total radical prostatectomy (%)	18.7 (236)	21.2 (216)	>0.05
Cystoprostatectomy (%)	9.8 (23)	26.9 (58)	<0.0001^a^
Incidental prostate cancer (%)	39.1 (9)	34.5 (20)	>0.05
Prostate cancer in prostatectomy (%)	78.4 (185)	70.4 (152)	>0.05
Age (year, mean ± SD)	58.7 ± 6.8 (185)	61.5 ± 7.4 (152)	0.0005
Gleason score (mean ± SD)	6.63 ± 1.1 (184)	6.58 ± 1.3 (149)	>0.05
Positive surgical margin (%)	33.5 (62)	19.7 (30)	0.0005^b^
Extracapsular extension (%)	18.9 (35)	17.8 (27)	>0.05
Seminal vesicle invasion (%)	9.7 (18)	8.6 (13)	>0.05
Lymph node invasion (%)	3.2 (6)	1.3 (2)	>0.05
Weight of prostate (grams, median)	46 (127)	45 (97)	>0.05
% of prostate gland (median)	15 (114)	6.6 (94)	0.00506
Volume (cm^3^, median)	7.4 (114)	3 (93)	0.00185

^a^: OR= 0.31, 95% CI: 0.18–0.52; ^b^: OR = 2.05, 95% CI: 1.243–3.38.

**Table 4:** Differences in bilaterally distribution of HGPIN, ASAP, and prostate cancer diagnosed at the first biopsy between African American and Caucasian.

	African American	Caucasian	*P* value
	*n/N*	*n/N*	
HGPIN	41/90 (45.6%)	25/75 (33.3%)	0.004^a^
ASAP	7/30 (23.3%)	1/21 (4.8%)	0.119^b^
Prostate cancer	295/479 (61.5%)	97/274 (35.4%)	<0.0001^c^

*n *= number of bilateral distribution; *N* = total cases with available information on pathology distribution; ^a^: Ratio = 2.51, 95% CI: 1.3–4.9; ^b^: Odds Ratio = 6.08, 95% CI: 0.6–143.3; ^c^: Odds ratio = 2.93, 95% CI: 2.1–4.
